# Within‐population variation in an invasive fish’ sociability when associating with conspecifics or heterospecifics

**DOI:** 10.1002/ece3.70118

**Published:** 2024-08-01

**Authors:** Morelia Camacho‐Cervantes, Alfredo F. Ojanguren

**Affiliations:** ^1^ Instituto de Ciencias del Mar y Limnología, Universidad Nacional Autónoma de México Mexico City Mexico; ^2^ Departamento de Biología de Organismos y Sistemas Universidad de Oviedo Oviedo Asturias Spain

**Keywords:** animal personality, behavioural syndromes, dispersal, heterospecific interactions, invasion success

## Abstract

Behavioural traits are key to promote invasion success because they are easier to adjust to changing environmental conditions than morphological or life history traits. Often, research has overlooked variance in behavioural traits within populations or has assumed it to be mere noise. However, a recent focus towards individual variation of behaviour of successful invaders has revealed new and more profound insights into the invasion process. Behavioural variation within a population could lead to more successful invasions, as they include individuals with diverse behaviours, which ensures at least some individuals could be able to cope with changing conditions. The aim of this research was to examine if invasive guppies (*Poecilia reticulata*) present within‐population differences in their sociability (time spent associating with a shoal) when interacting with conspecifics or heterospecifics. Guppies presented significant differences in their individual tendencies to associate with conspecific or heterospecific shoals. There were among‐individual differences in the time spent shoaling with conspecifics versus heterospecifics, where most individuals did not differ in their sociability with conspecifics or heterospecifics, and only 22% of individuals presented a higher tendency to associate with conspecifics. Our results are the first to show individual differences in fish’ tendencies to associate with heterospecifics among individuals of the same population and rearing conditions. Given that associations with heterospecific natives have been found to be as beneficial as associations with conspecifics for invaders, our results contribute to the understanding of mechanisms behind heterospecific sociability between natives and invaders.

## Graphical abstract

We examined sociability in guppies, demonstrating within‐population differences in sociability, which could have broader implications for invasion ecology. By revealing differences in associations with heterospecifics among individuals, the study sheds light on the complex mechanisms behind interactions between native and invading species.

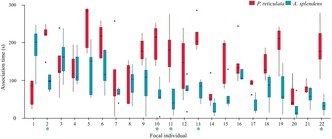

## 1 INTRODUCTION

Invasive species pose a significant threat to biodiversity and cost millions of dollars annually to the global economy (Diagne et al., 2021). Behavioural traits of non‐native individuals are central to invasion success because they allow individuals to adjust quickly to novel environments (Wong & Candolin, 2015). Successful invasive species tend to have flexible behavioural responses, and this can be achieved through individual plasticity, or different kinds of individual responses that are consistent over time and contexts (Chapple et al., 2012; Reale et al., 2007). The study of animal personality has the potential to enhance our understanding of the traits pertaining to successful invasions (Collins et al., 2023; Juette et al., 2014). Indeed, Wolf and Weissing (2012) highlight that animal personality is a key ecological driver that should be considered when researching invasion dynamics.

Populations with different behaviours among their members are thought to be more successful at invading new ecosystems with very different environmental conditions compared to their original habitat (Fogarty et al., 2011). Populations with among‐individual variation in behaviour might include individuals with traits that favour dispersal or establishment (Cote et al., 2012; Fogarty et al., 2011). For example, some individuals can promote dispersal of a population by presenting a higher tendency to explore novel environments, increasing predation risk, but also the chances of finding new food sources or other valuable resources (Luttbeg & Sih, 2010; Rands et al., 2003). Behavioural variation among individuals of a population might increase its invasion success as less social individuals could avoid high‐density areas, facilitating dispersal, while social individuals are attracted to high population densities, promoting cohesion and the establishment of an invading population (Fogarty et al., 2011). Identifying species that present higher among‐individual variation in their behaviour and understanding these behaviours in the context of invasion could improve the effectiveness of management, as these species could be considered at higher risk of becoming successful invaders (Courchamp et al., 2017; Wolf & Weissing, 2012).

Invasive organisms typically occur at low densities during the initial stages of invasion which can be detrimental for fitness as they might be subject to Allee effects – disadvantages of being part of a small population, such as lower efficiency to locate food, refuges or mating partners (Tobin et al., 2011). High sociability towards native heterospecifics can help overcome some of these Allee effects (Camacho‐Cervantes et al., 2023). Individuals that are more willing to associate with other animals can get the benefits (*e.g*., predator avoidance, foraging success) of being part of a larger group even if the group is composed of heterospecifics (Hernández‐Brito et al., 2021; Paijmans et al., 2020; Santiago‐Arellano et al., 2021). Thus, heterospecific interactions can potentially increase the fitness of invasive species (Wyatt et al., 2013).

The Trinidadian guppy (*Poecilia reticulata*) is a widespread invasive freshwater fish, with populations established in every continent except Antarctica (Deacon et al., 2011). In Mexico, invasive *Poeciliids* have been shown to increase the pressure upon endangered endemic goodeids, including the extinct‐in‐the wild *Zoogoneticus tequila* (Suárez‐Rodríguez et al., 2023). Previous research has demonstrated that guppies show a tendency to associate with other species even if they do not share evolutionary history; when presented with the opportunity to shoal with goodeids guppies show a tendency to associate with them rather than remaining alone (Camacho‐Cervantes et al., 2018; Camacho‐Cervantes, Macias, et al., 2014; Camacho‐Cervantes, Ojanguren, et al., 2014). These associations are beneficial to guppies as they acquire information from natives and become keener to explore novel environments (Camacho‐Cervantes et al., 2015). Indeed, heterospecific interactions result in direct benefits, for example guppies are able to locate food faster and spend more time foraging by increasing the shoal size with natives (Camacho‐Cervantes, Macias, et al., 2014). We know that guppies show a higher tendency to associate with conspecifics over heterospecifics (Camacho‐Cervantes, Ojanguren, et al., 2014), but it is still uncertain whether this applies to all individuals in the population or if certain individuals show a different pattern.

The aim of this study is to evaluate among‐individual variation in guppies' tendency to shoal with conspecifics or heterospecifics. By testing individual guppies several times with each type of shoal, we will also be able to test the idea that some individuals are more social with conspecifics than with heterospecifics and vice versa. We hypothesise that individual guppies within the same population show varying tendencies to associate with a given shoal. Behavioural differences among individuals have also been found in *Gambusia affinis*, a poeciliid with a biology similar to guppies and a successful worldwide freshwater invader (Cote et al., 2010). Behavioural plasticity among individuals could help overcome Allee effects during the early stages of the invasion process, or promote dispersal once established contributing to the guppy's invasive spread (Camacho‐Cervantes et al., 2023).

## 2 MATERIALS AND METHODS

Experiments took place at The University of St Andrews, Scotland, UK, during October and December 2013. We used adult female guppies (*P. reticulata*) and juvenile butterfly splitfins (*Ameca splendens*) from laboratory stocks. Butterfly splitfins are goodeids native to the Mexican Central Plateau that have a similar phenotype and share habitat and ecological requirements with other species of poeciliids (Gesundheit & Macías, 2018). In fact, both species have been collected at the same time in sites where guppies are invasive (MCC, unpublished data). We used only females as they allocate more time to social interactions, while males often engage in mating behaviour harassing both female guppies and heterospecifics (Magurran, 2005; Valero et al., 2008). Experimental fish were descendants from wild individuals collected in their native habitats (guppies in lower Tacarigua River, Trinidad, splitfins in the headwaters of Teuchitlan, Mexico). All fish were kept in aquarium stock tanks (45 × 30 × 30 cm), each containing 15–20 fish. Tanks contained aged tap water, a filter to maintain water quality, plants, and river gravel. Water temperature ranged between 20 and 26°C. A 12‐h light and 12‐h dark photoperiod was maintained from 08:00 to 20:00 h. Fish were fed commercial flake food, Tetramin®, every morning at least 30 min before the first recording took place to avoid food‐searching behaviour or hunger. By doing this, we ensured fish were not hungry (since they usually are fed once a day) and no longer engaged in feeding behaviour.

Experiments were conducted daily, between 10:00 and 12:00 h. Since fish were not tagged, and to keep track of their identities, 22 focal female guppies were placed in separate tanks (20 × 30 × 30 cm) from the start to the end of the observations. They were able to see individuals in neighbouring tanks to minimise the effect of isolation, as fish were able to see at least two neighbouring fish, they had at all times other fish to interact with. Shoals were composed of three fish that were kept in a stock tank for at least 2 weeks prior to the experiment to avoid familiarity with focal individuals (Griffiths & Magurran, 1997). The shoal pools consisted of 53 female guppies and 32 juvenile butterfly splitfins, from which the three fish that were to compose the stimulus shoal were selected haphazardly from different stock tanks to avoid pseudoreplication (Hurlbert, 1984).

**FIGURE 1:**
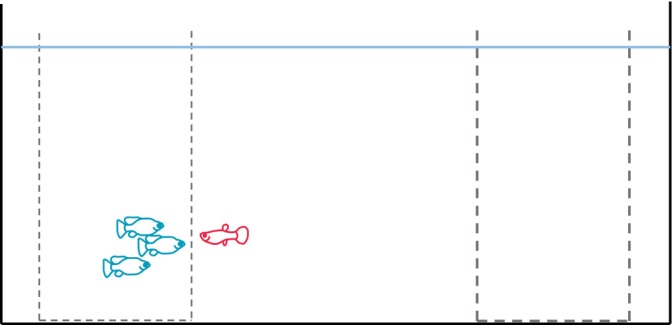
Diagram of the experimental tank setup. Association was recorded whenever the fish was within one body length of the bottle containing a shoal.

Experiments were carried out by a single observer, following previously established methodology (Camacho‐Cervantes et al., 2018; Camacho‐Cervantes, Ojanguren, et al., 2014; Salazar‐Rueda et al., 2024), in four adjacent, visually isolated experimental tanks (45 × 30 × 30 cm) equipped with one central plastic container to isolate the focal individual from the shoal and their olfactory cues. Prior to data collection, this container was gently lifted to release the focal at the beginning of each observation. Two plastic bottles were placed at both ends of the tank; one containing the shoal and one empty in case focal fish reacted to an object (the bottle) and not the presented shoal. Shoals were placed in one or the other bottle randomly. The bottles were perforated to allow circulation of water and chemical cues between the bottles and the tank (Figure 1). The shoal species composition (conspecifics or heterospecifics) and order of focal fish testing were randomised throughout the experiment. Fish were acclimated (~5 min) to the new environment and did not exhibit signs of stress before data collection.

Tendency to associate with the presented shoal was measured as the total time (in seconds) spent within one body length (~20 mm) of the bottle containing a shoal over a 5‐min period (Figure 1). It is conservative to consider that fish were associating with others when within one body‐length distance of the bottle, because our visual observations suggest they could even be moving in a coordinated way even from a larger distance. Each focal individual was tested a total of 10 times over 10 consecutive days, five times with each shoal type (conspecific and heterospecific) in a haphazardly way to produce an estimate of their individual behaviour. Fish were tested only once a day. All fish to be used in this experiment were selected to be approximately the same size, they were on average 21 mm (SD = 2.5). Still, after the observations, fish were placed in a Petri dish with a small amount of water and photographed from above to measure standard length using Image‐J software (Rasband, 2015). We calculated the difference between the focal fish and the average size of the shoal and included it in the analysis.

### 2.1 Statistical analysis

To test for differences in individual sociability (seconds associated) towards heterospecifics or conspecifics, a linear mixed effects model (lme), from the ‘nlme’ R package (Pinheiro et al., 2019), was conducted including shoal species, the interaction between shoal species and focal individuals, and the difference in size between the focal and the shoal as fixed factors. We included focal individual as a random factor to account for within‐subject correlation, considering our repeated measures approach. The distribution of the residuals and homogeneity of variances for our model were confirmed to be normal, by plotting its Residuals vs. Fitted Plot, Q–Q plot, and a Density Plot of Residuals (Figures S1–S3). As a post‐hoc test, for each focal, we compared the difference in association time they showed for conspecifics and heterospecifics using a Kruskal‐Wallis test. To account for multiple comparisons in the post‐hoc analysis, we use the Benjamini‐Hochberg procedure using an alpha of 0.05 as the significance threshold (Benjamini & Hochberg, 1995). Statistical analyses were carried out using R‐console® (R Core Team, 2019).

## 3 RESULTS

**FIGURE 2:**
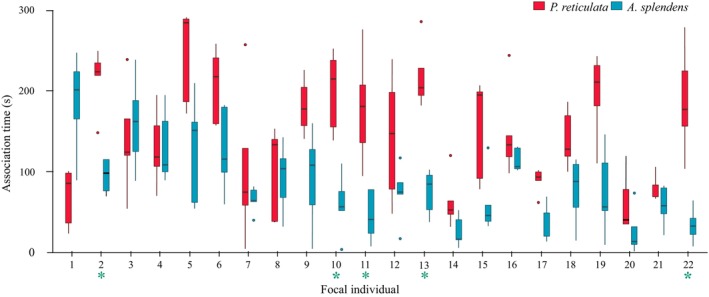
Association time of focal guppy individuals interacting with shoals of conspecifics (red bars) and heterospecifics (blue bars). Green asterisks under focal individual number mean fish showed a significantly higher tendency to associate with conspecifics.

There was an interaction between focal individuals and the species of the shoal presented to them (lme, *F*_42,155_ = 2.7, *p* < .001, Figure 2). In general, focal individuals spent different amounts of time associating with conspecific or heterospecific shoals (lme, *F*_1,155_ = 99.7, *p* < .001, Figure 2), and the difference in size between the focal and the shoal had no effect in their sociability (lme, *F*_1,155_ = 0.3, *p* = .61, Figure 2). However, our post‐hoc analysis, after the Benjamini‐Hochberg correction, revealed five fish presented a significantly higher tendency to associate with conspecifics, while most fish (17) did not show differences in their tendencies to associate towards conspecifics or heterospecifics. No fish associated significantly longer with heterospecifics than conspecifics.

## 4 DISCUSSION

Populations displaying among‐individual variation in behaviour are considered to show greater success when invading novel environments (Fogarty et al., 2011). In this study, we demonstrate that within a population, there were among‐individual differences in guppies' tendency to shoal longer with either con‐ or heterospecifics. Our findings agree with research investigating other important poeciliid invasive species – such as the mosquitofish and guppies, which also present individual differences in their tendencies to associate with other fish (Cote et al., 2010; Irving & Brown, 2013). Many studies investigating sociability traits, such as Fogarty et al. (2011) or Cote and Clobert (2007), refer to bimodal sociability in terms of ‘social’ or ‘asocial’ individuals. However, at least in guppies, sociability is a continuous variable (Camacho‐Cervantes et al., 2018). Populations are likely to consist of individuals that are relatively more or less social within a continuum, rather than distinct groups.

At the species level, guppies are more sociable than native goodeids and other native poeciliids, both when the group of fish available to shoal was composed of other guppies or fish from other species (Camacho‐Cervantes et al., 2018). However, our results show there was within‐population variability in individual's tendency to shoal longer with conspecifics or heterospecifics. Heterospecific interactions between native and non‐native species at the individual level that could lead to benefits for non‐natives have been seldom studied (Camacho‐Cervantes et al., 2023). Heterospecific interactions could present considerable advantages to invasive species because they could avoid Allee effects (i.e., disadvantages of being part of a smaller population) (Camacho‐Cervantes et al., 2023) and establish new populations. The benefits of associating with heterospecific individuals range from being able to grow faster (Nelson et al., 2017; Sardiña et al., 2015) to avoiding predators (Gestoso et al., 2014). However, associating with heterospecifics could be posing a toll to species as these interactions also result in disadvantages, such as parasite transmission or resource sharing (Krause et al., 2002). The fact that most of our focal fish (77%) did not differ in their tendencies to associate with conspecifics or heterospecifics enables them to join heterospecific shoals when there are few conspecifics available, and by doing so deriving the benefits of being in a group (Camacho‐Cervantes et al., 2015; Camacho‐Cervantes, Macias, et al., 2014). On the other hand, since some individuals presented a higher tendency to associate with conspecifics, these individuals could be the ones enhancing conspecific grouping.

Sociability is suggested to enhance the spreading and establishment of invading populations through density‐dependent dispersal (Fogarty et al., 2011). There is growing evidence indicating that behavioural trait differences within a population can influence the dispersal patterns of animals. Indeed, research carried out on the invasive signal crayfish (*Pacifastacus leniusculus*) reveals that individual traits had both positive and negative effects on dispersal, while population density and the complexity of the local habitat were also significant factors in determining dispersal patterns (Galib et al., 2022). Our research shows that most guppy individuals do not present a higher tendency to associate with conspecifics over heterospecifics, still some of them do. We hypothesise that, during the establishment and dispersal stages of invasion, individuals that do not show differences in their tendencies to associate with conspecifics or heterospecifics may be the ones promoting invasion success. These individuals would be the ones associating with available shoaling mates and deriving the benefits from doing so through heterospecific associations with native species (Camacho‐Cervantes et al., 2023). Once the establishment phase is over, individuals that show a higher tendency to associate with conspecifics could be further promoting the invasion success of the populations as the benefits associated with grouping would remain among conspecifics.

Heterospecific sociability and individual behaviour are important to the dispersal and establishment of an invading population (Cote et al., 2010). Dispersal tendency in invasive species has been linked to boldness (Dingemanse et al., 2003; Fraser et al., 2001) or experience (Barbosa et al., 2016). Individual differences could also be of importance during the dispersion stage of the invasion process, particularly as in other aquatic invasive species it has been linked to dispersion even in fragmented habitats (Daniels & Kemp, 2022) or under climate change scenarios (Zhao & Feng, 2015). Explicitly considering these traits in models predicting the speed and success of invasions could help identify potentially successful invaders. By recognising these invaders early, we can prevent them from establishing in new environments or manage them during the initial stages, when their impacts are still manageable and less severe (Courchamp et al., 2017).

## AUTHOR CONTRIBUTIONS

**Morelia Camacho‐Cervantes:** Conceptualization (equal); data curation (equal); formal analysis (equal); investigation (equal); methodology (equal); project administration (equal); resources (equal); validation (equal); visualization (equal); writing – original draft (equal); writing – review and editing (equal). **Alfredo F. Ojanguren:** Conceptualization (equal); funding acquisition (lead); investigation (equal); methodology (equal); project administration (equal); resources (equal); supervision (lead); validation (equal); visualization (equal); writing – original draft (equal); writing – review and editing (equal).

## CONFLICT OF INTEREST STATEMENT

Authors declare no conflict of interest.

## Supporting information


Data S1.

Data S2.

Figure S1.

## Data Availability

Data and script of analysis are included as Data S1 and S2.
