# Supplementary material for: Within‐population variation in an invasive fish’ sociability when associating with conspecifics or heterospecifics
Source: Ecol Evol. 2024 Aug 1;14(8):e70118. doi: 10.1002/ece3.70118 (PMC11293883; doi:10.1002/ece3.70118)
Supplement: Supplementary file 3 — Figure S1. [file ECE3-14-e70118-s001.docx]

Supplementary Material for:

**Within population variation in an invasive fish’ sociability when associating with conspecifics or heterospecifics**

Morelia Camacho-Cervantes, Alfredo F. Ojanguren

**Distribution of the residuals and homogeneity of variances for our model (lme)**

**Figure 1. Residuals vs. Fitted Plot**


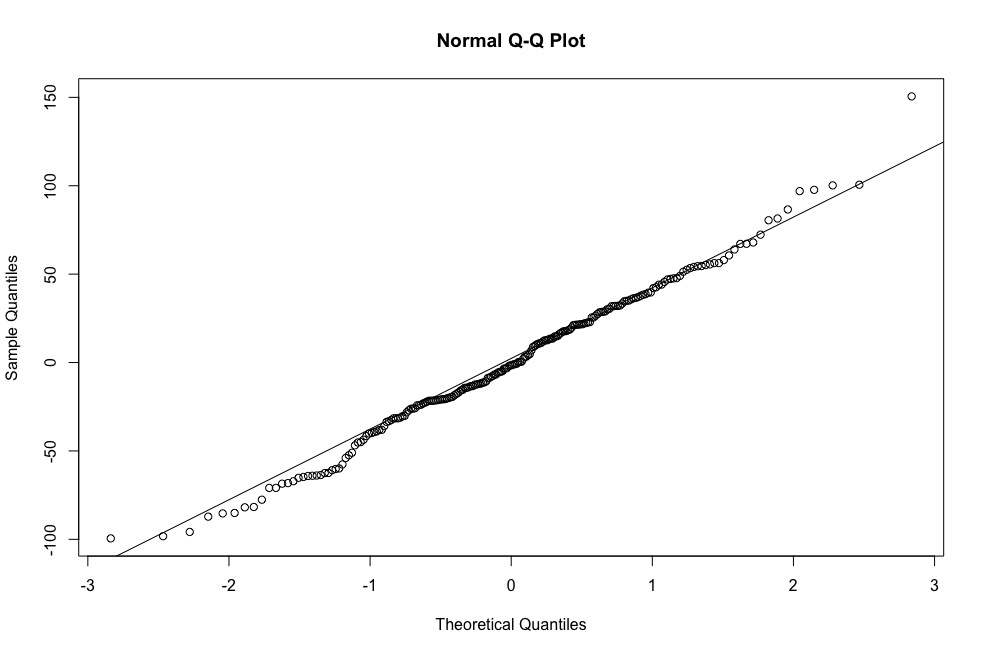
**Figure 2. Quantile – Quantile Plot**


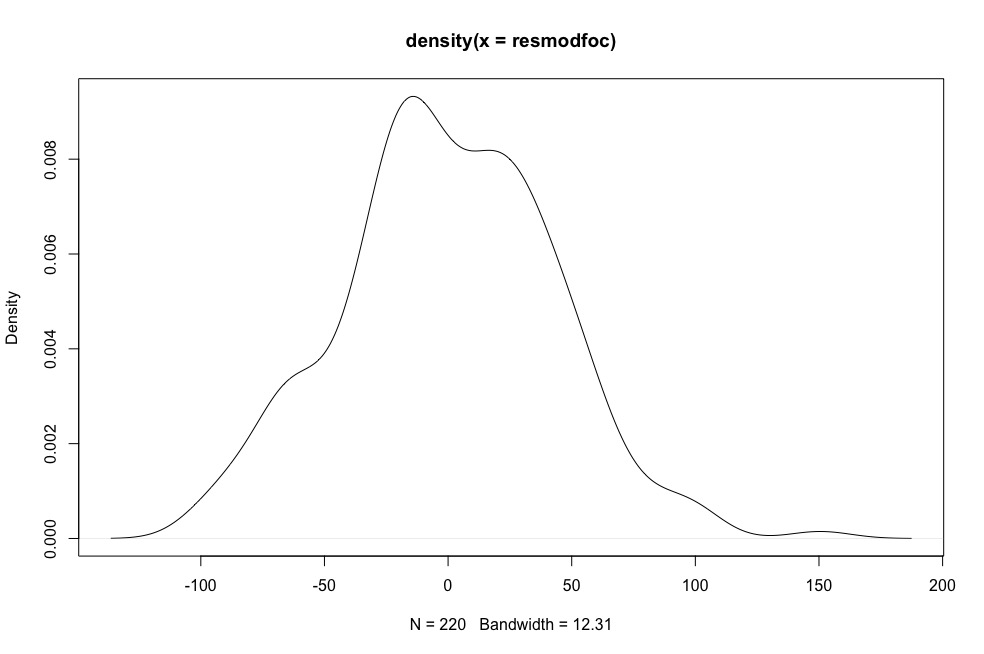


**Figure 3. Density Plot of Residuals**
